# Preclinical Validation of MIN-T: A Novel Controlled-Released Formulation for the Adjunctive Local Application of Minocycline in Periodontitis

**DOI:** 10.3390/antibiotics13111012

**Published:** 2024-10-28

**Authors:** Małgorzata Benedyk-Machaczka, Piotr Mydel, Karsten Mäder, Marta Kaminska, Nadine Taudte, Marcel Naumann, Martin Kleinschmidt, Sandra Sarembe, Andreas Kiesow, Sigrun Eick, Mirko Buchholz

**Affiliations:** 1 H&G Ltd., 31-431 Krakow, Poland; malgorzata.benedyk@uj.edu.pl (M.B.-M.);; 2 Department of Microbiology, Faculty of Biochemistry, Biophysics and Biotechnology, Jagiellonian University, 30-387 Krakow, Poland; 3 Institute of Pharmacy, Martin Luther University Halle-Wittenberg, Kurt-Mothes-Str. 3, 06120 Halle (Saale), Germany; karsten.maeder@pharmazie.uni-halle.de; 4 PerioTrap Pharmaceuticals GmbH, Weinbergweg 22, 06120 Halle (Saale), Germany; 5 Department Drug Design and Analytical Chemistry, Fraunhofer IZI-MWT, Weinbergweg 22, 06120 Halle (Saale), Germany; marcel.naumann@izi.fraunhofer.de (M.N.);; 6 Department Biological and Macromolecular Materials, Fraunhofer Institute for Microstructure and Systems IMWS, Walter-Huelse-Strasse 1, 06120 Halle (Saale), Germany; 7 Department of Periodontology, School of Dental Medicine, University of Bern, Freiburgstrasse 7, CH-3010 Bern, Switzerland; sigrun.eick@unibe.ch

**Keywords:** periodontitis, proof-of-concept, adjunctive therapy, minocycline, local application, controlled release, non-clinical, resistance, biodegradation

## Abstract

**Background:** Adjunctive treatment of periodontitis lacks solutions which allow for enough time for wound healing in the periodontal pockets by avoiding fast re-colonization. Such a solution might be an antibiotic-containing formulation with a controlled release over a period of weeks. Here, a recently described minocycline-containing approach is qualified for further clinical development by focusing on proof-of-concept, systemic burden, resistance development, and degradation studies. **Methods:** Animal studies were done in two different (mouse-chamber, rat *Porphyromonas gingivalis* challenging) models, including effects on inflammation markers, bone loss, and bone structure. Also, serum concentrations of minocycline after local application were determined by HPLC-MS/MS. The resistance status of bacterial clinical isolates against minocycline was investigated and the degradation of the formulation was characterized by laser scanning and scanning electron microscopy. **Results:** Animal studies clearly demonstrated the applicability of the new formulation in the investigated models. Inflammation markers decreased in a dose-dependent manner and reduced bone loss compared to non-treated group was observed. Therefore, the systemic burden of the antibiotic was neglectable. Minocycline is still effective against oral pathogens; resistance development was not seen. The biodegradable thread was first swollen and subsequently degraded over a period of weeks. **Conclusions:** The results support the continued clinical development of this new formulation. A phase I clinical trial is planned to further evaluate its safety and efficacy.

## 1. Introduction

Periodontitis is a chronic inflammatory and destructive disease of the tooth-surrounding tissue which can lead to tooth loss. It is associated with a shift of an eubiotic to a dysbiotic microbiota within the plaque biofilm [1,2]. The most significant periodontopathogens in the pathogenesis of periodontitis include *Porphyromonas gingivalis*, *Tannerella forsythia*, and *Treponema denticola*. These obligate anaerobes thrive in the protein-rich environment of the deep periodontal pockets by modulating the host’s immune response. Without treatment, sustained inflammation can lead to the destruction of both soft and hard tissues, ultimately resulting in tooth loss [1].

Despite the medical advances made in recent years in the fields of antimicrobials and other periodontal treatments, the prevalence of periodontitis in the adult dentate population is estimated to be more than 60% with severe cases at 23.6% [3,4]. Thereby a successful treatment with the aim of controlling the disease in an acceptable state depends strongly on patient compliance, the severity of the disease itself, and other individual risk factors, e.g., smoking, diet, and the existence of other diseases [5,6,7]. Due to the association of periodontitis with several systemic conditions [8,9,10], the healthcare costs generated by this disorder are significant and underline the need for novel and more efficient treatment and maintenance strategies [11]. The current standardized periodontal therapy is performed stepwise, the first step focuses on supragingival biofilm removal and risk factor control, the second step (cause-related therapy) includes subgingival biofilm and calculus removal [5]. Among others, locally administered antibiotics might be considered adjuncts to instrumentation, whereas the use of adjunctive systemic antibiotics may only be considered in young patients with generalized periodontitis stage III [5,12].

The use of systemic antibiotic therapy in severe periodontitis treatment has been found to result in improved clinical attachment gain and the reduction of probing depth [13]. The most applied is the combination of amoxicillin and metronidazole [14,15]. That, coupled with the high oral dose administration necessary to reach minimum inhibitory concentrations of the antibiotics in the periodontal pockets, is often associated with systemic side effects [16]. It is estimated that more than one in twenty-five patients treated with systemic antibiotic therapy will experience one of the following: allergic reactions, gastrointestinal problems, central nervous system problems, nephritis, and among other symptoms. About 27% of patients treated with doxycycline have experienced nausea. Furthermore, systemic antibiotic usage contributes to antibiotic resistance, which at the current rate progresses faster than the development of novel antibiotics [16].

In contrast, topical antimicrobials used in periodontal therapy allow for the bypassing of these issues [17,18,19]. Antibiotics can be applied as in situ forming implants, gels (Atridox^®^, containing doxycycline, Ligosan^®^, containing doxycycline), or preformed microspheres (Arestin^®^, containing minocycline) that are placed directly into the periodontal space. Depending on the polymers used as vehicles in such treatment, antibiotics are steadily released directly into the inflamed area over a period of days to up to three weeks. Among the commercially available products, Actisite^®^ (removed from the market), ethylene/vinyl acetate polymer fibres releasing tetracycline hydrochloride [20], were placed into periodontal pockets for 10 days, have been shown to benefit patients only marginally, and required mechanical removal after the treatment period [19,21]. In contrast, while self-degrading delivery systems containing doxycycline (Atridox^®^) or metronidazole (Elyzol^®^) offer patients in principle greater benefits in controlling pathogen growth and the clinical symptoms, their applications necessitate the use of specialized equipment or skills. Unfortunately, Atridox^®^ as well as Elyzol^®^ are no longer marketed in the European Union. By far the most promising adjunct topical treatment currently available on the market (only in the U.S.) is Arestin^®^, microspheres releasing minocycline hydrochloride, that has been shown to release the antibiotic for about 21 days in vitro [22]. And it can significantly improve patient benefits (such as reducing pocket depth or bleeding on probing) when compared to scaling and root planing (SRP) alone [23,24,25]. Application of Arestin^®^ also requires specialized equipment, but clear benefits provided by minocycline treatment warrant further optimization of antibiotic carriers to simplify the treatment procedures. Despite the much lower risks for systemic side effects, locally applied antibiotics also might lead to unwanted actions. For Arestin^®^, several possible adverse events are described, which include, e.g., tooth disorders, dental pain, headache, pharyngitis, etc. [26].

Recently, we reported on the technological development of a novel pharmaceutical composition (minocycline-threads, MIN-T) containing minocycline in a defined complex with magnesium stearate (minocycline lipid complex, MLC) [27,28]. This new formulation is characterized by a long in vitro release of 42 days, which was microbiologically proven in a model simulating the flow of gingival crevicular fluid [22]. All in all, MIN-T combines several advantages: it doubles the time of controlled release in vitro as compared to Arestin^®^, it is biodegradable (no removal by the dentist necessary), and is easily applicable as a flexible thread into the periodontal pockets with dimensions of 0.6 mm in thickness and 4 mm in length. To further characterize this completely new formulation before transferring into the clinics, MIN-T was characterized in a battery of different in vitro as well as in vivo tests, which are presented in the following. Thereby, the degradation of MIN-T was investigated using laser and electron scanning microscopy. The susceptibility pattern and the potential development of resistance against the active pharmaceutical ingredient minocycline were determined for clinical isolates of relevant periodontal pathogens. Finally, the in vivo efficacy as well as the tolerability and the systemic pharmacokinetic properties of MIN-T were tested after a local application in two animal models.

## 2. Results

### 2.1. Generation of the MIN-T

The production of the test material was done as described elsewhere [27], whereas the resulting threads contained 11.5% (*m*/*m*) of minocycline, calculated based on the base of the antibiotic. The quality of the testing material used was completely in line with the results from the pharmaceutical development process and compliant with the predefined parameters.

### 2.2. Swelling and Degradation of MIN-T

The swelling behaviour and degradation of the MIN-T extrudates were investigated while storing them in phosphate buffered saline (PBS) at 37 °C for up to 38 days.

**Figure 1:**
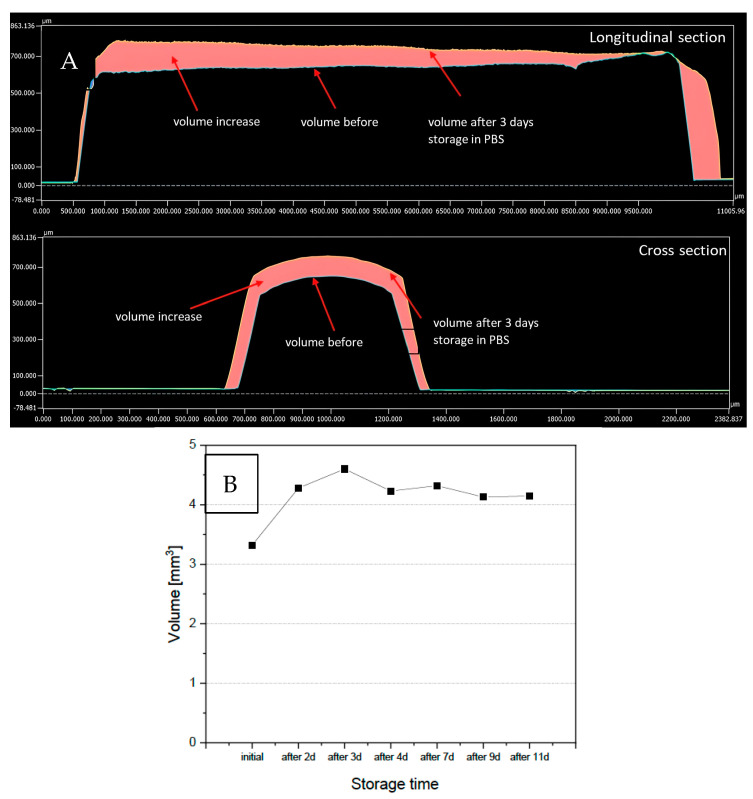
Swelling behaviour of the extrudates: (**A**) analyzed volume increase before and after 3 days storage of the bioresorbable extrudate; (**B**) calculated volume of the bioresorbable extrudate before and after different storage times in PBS.

The swelling behaviour (increase in volume and size) was followed for 11 days by using laser scanning microscopy (LSM). Thereby, the highest increase in volume was observed after 3 days. The measured volume enlarged by more than 40% compared to the starting volume, leading to an increase of the radius from approx. 600 µm to approx. 660 µm. Between days 4 and 11, the volume of the thread remained relatively constant which corresponds to an increase in the volume of nearly 30% compared to the original volume (Figure 1).

**Figure 2:**
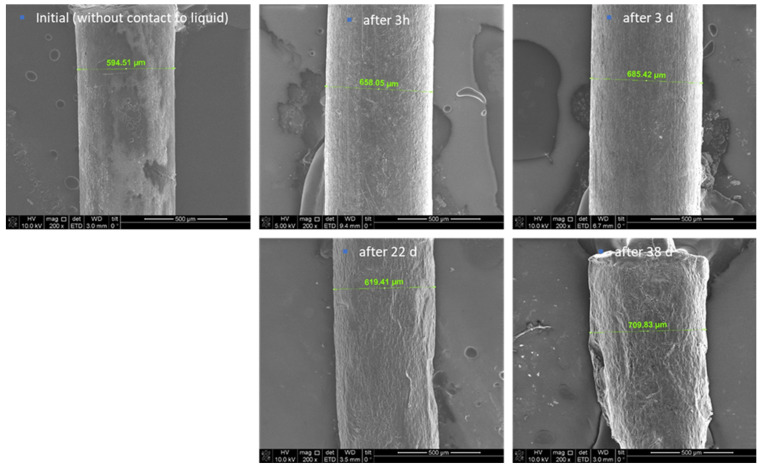
SEM images of the extrudates before, after 3 h, 3 d, 22 d, and 38 d of storage in PBS, 200× magnification.

Furthermore, scanning electron microscopy (SEM) images were depicted and used for the description of the degradation behaviour of the formulation. Thereby, the initial state of the formulation is characterized by an inhomogeneous, partly rough surface (Figure 2). Structures with crystallite-like shapes are clearly visible. This finding can be explained by the included magnesium stearate of the MLC, which is known to form such surface structures. Again, an increase in the thickness of the thread and thus a clear swelling was determined after 3 h and 3 days in PBS, respectively. Even after 22 days the thread diameter was still larger compared to the initial situation; however it was smaller than after 3 days. A possible explanation might be that fragments start to detach from the surface, which is underlined by the detection of small amounts of magnesium stearate in the daily exchanged PBS. The ongoing degradation results in the formation of very small pieces, so after day 38 it was not possible to generate samples for further investigation of the process.

**Figure 3:**
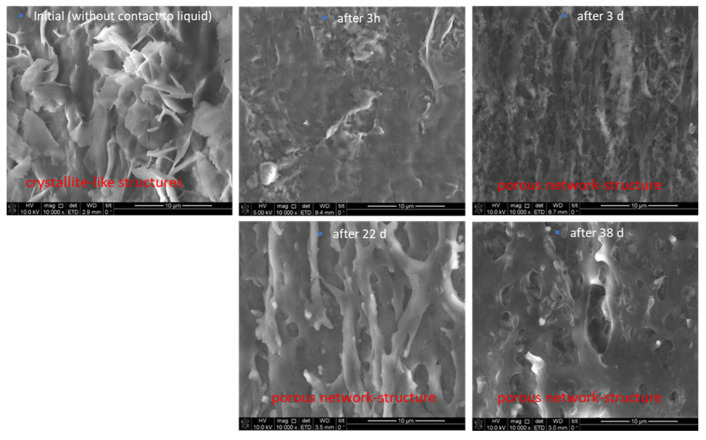
SEM images of the extrudates before, after 3 h, 3 d, 22 d, and 38 d storage in PBS, 10,000× magnification.

At higher magnifications (Figure 3), cracks and break-off edges were visible starting on day 22, which are hints for the progressing dissolution and degradation process. A porous network structure became more and more visible after a prolonged storage time in the buffer. As described above, after 38 days, the polymer extrudate was broken into several smaller pieces. Analysis of the pieces suggested a repeated swelling on the one hand but, on the other hand, the fact was that PBS was not penetrating through the complete extrudate before. After 38 days, ongoing storage of the formulation was stopped due to its progressing degradation, which made it impossible to further investigate the process.

### 2.3. Potential Development of Resistance

A potential development of resistance against minocycline (minocycline hydrochloride) was studied in 11 clinical isolates (3× *P. gingivalis*, 4× *F. nucleatum*, 4× oral *streptococci*) obtained from different patients with periodontitis. This investigation was done following already described methods [29,30,31]. The strains were passaged under the pressure of subinhibitory minimal inhibitory concentrations (MIC) of minocycline up to 50 passages. Before and after every 10 passages, current MICs were determined.

For *S. gordonii*, *S. constellatus*, *S. mitis*, *S. oralis*, and *P. gingivalis*, a relevant increase in the MIC was not found in up to 50 passages. Only some variabilities for one order of magnitude were seen for *S. mitis* and for one *P. gingivalis* strain, but without showing a gradual increase in the values. This was different for *F. nucleatum*. Here a few strains were lost over the passages, due to experimental issues. In contrast to all other strains, the clinical isolate BeOR1 showed a clear increase in MIC after 20 passages and resulted in a four times higher MIC of 0.250 µg/mL compared to the start with 0.016 µg/mL. Interestingly, this increase was not a trend in the following experiments. The higher value was stable for the next 30 passages and did not further increase (Table 1).

### 2.4. In Vivo Efficacy—Mouse Chamber Model

Following already described procedures [28], titanium chambers were surgically implanted subcutaneously into the backs of specific pathogen-free (SPF) 8–12-week-old BALB/c mice (dorsolumbar region), after placing MIN-T according to six different dosages (0 (control group), 1, 10, 25, 40, 80 mg/kg body weight) into the chambers. Thereby, each group contained seven animals. After the complete healing of the incisions, 2 × 10^8^ *P. gingivalis* W83 was injected into the lumen of the chamber. Chamber fluids were analysed for levels of minocycline by HPLC-based methods.

**Figure 4:**
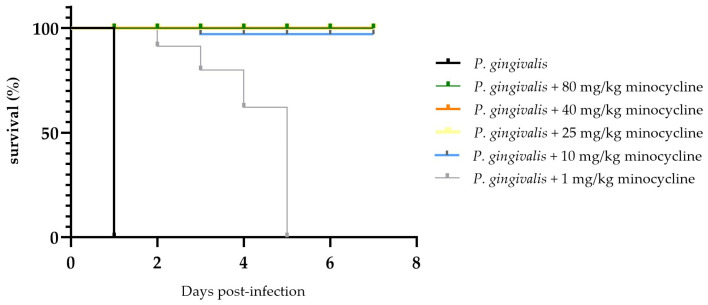
Survival curve of the animals after treatment with a lethal dosage of *P. gingivalis* into the chamber. A clear dose related effect is visible. Without any treatment animals died nearly immediately after application. Except for 1 mg/kg for all applied dosages, the animals survived until the end of the experiment.

A clear dose response to MIN-T was observed regarding the mortality of the animals. In the control group, all seven animals succumbed within 24 h following inoculation with *P. gingivalis* (strain W83). Conversely, animals treated with MIN-T exhibited dose-dependent protection. The effective concentration ranged from 1 mg/kg to 80 mg/kg, with varying levels of efficacy. At higher doses (80, 40, 25 mg/kg), the formulation completely prevented pathogen-induced mortality. A reduction in the dose to 10 mg/kg led to the death of one animal on day 3, while the lowest tested dose (1 mg/kg) only delayed the onset of symptoms related to *P. gingivalis* dissemination, with all animals in this group dying by day 5 of the experiment (Figure 4).

**Figure 5:**
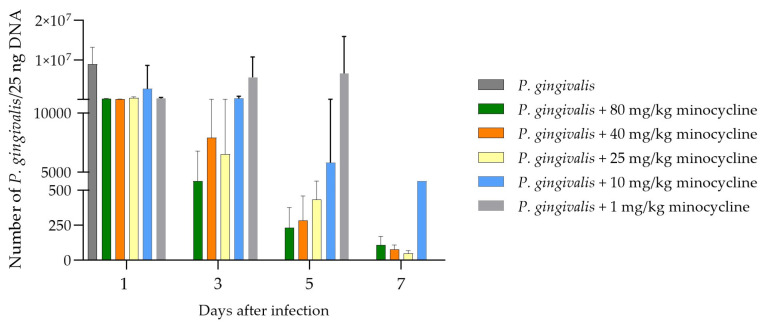
No. of *P. gingivalis* DNA after application of a lethal dosage of *P. gingivalis* into the chamber, filled with different dosages of the novel formulation, different days after application. Again, a clear dose-dependent effect is visible and correlates nicely with the figure above. Except for the dosage of 1 mg/kg minocycline, a strong reduction in the pathogen DNA is demonstrated.

Chamber fluid sampled on days 1, 3, 5, and 7 was analyzed to assess the growth rate of *P. gingivalis*. Bacterial DNA extracted from the fluid underwent qPCR analysis (Figure 5). Within the first 24 h, a significant reduction in bacteria was observed across all treated groups. However, the group receiving the lowest dose of 1 mg/kg exhibited a steady increase in bacterial counts over the next 48 h, leading to delayed mortality. Animals treated with 25, 40, and 80 mg/kg of minocycline showed very low levels of *P. gingivalis* by day 3, resulting in complete pathogen elimination by day 7. Those treated with 10 mg/kg experienced a gradual decrease in *P. gingivalis* counts throughout the experiment, though the decline was slower compared to higher doses. This resulted in significantly higher *P. gingivalis* counts at the end of the experiment relative to the 25, 40, and 80 mg/kg groups. Despite persistent infection, no mortality was observed in the 10 mg/kg group, and the overall health of these animals remained satisfactory. As expected in the 1 mg/kg group, only a small reduction of the bacterial count was found on day 1 compared to the control group. After this the amount steadily increased and was on the same level on days 3 and 5 as the control on day 1. Due to the mortality of the animals in that group, no data are available for day 7.

**Figure 6:**
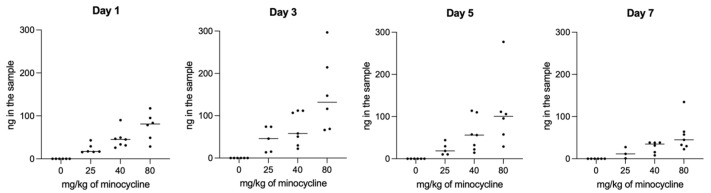
Visualization of the minocycline concentration after the placement of different dosages of the novel formulation MIN-T into the chambers. Days start to count after application of *P. gingivalis* into the chamber, so Day 1 is the amount of minocycline after the placement of the chamber and healing for 10 days (11 days after application).

The concentrations of minocycline in the chambers as measured by HPLC (Figure S1) were stable throughout the whole experiment and increased in line with the applied dosage (Figure 6). This highlights the steady release of the antibiotic throughout the whole experiment.

All surviving animals were sacrificed on day 7 and the blood was collected for the determination of systemic levels of minocycline and inflammatory biomarkers.

To determine the systemic concentration of minocycline, serum samples were analyzed using LC-MS/MS. In none of the samples, the minocycline concentration exceeded the lower limit of quantification (LLOQ) of 20 ng/mL, which was established for mouse serum analytics during method development (Table S1). Despite being below the LLOQ, the data exhibit a clear dose-dependent trend in serum levels, with the highest administered dose (80 mg) yielding the highest systemic concentration of minocycline (range: 5.0–10.8 ng/mL). For the lowest dosages of 1 mg/kg and 10 mg/kg, however, determination of minocycline levels in the serum was not possible.

**Figure 7:**
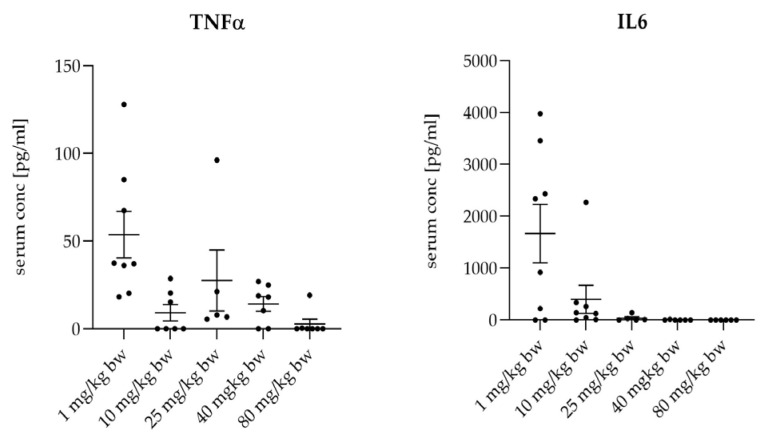
Determination of the serum level of three inflammation markers after placement of different dosages of the novel formulation and the application of a lethal dose of *P. gingivalis*. Data are collected after the animals were sacrificed. For all marker molecules, a dose dependency is visible, whereas for the lowest dosage of 1 mg/kg, the inflammation markers have the highest level.

The treatment with MIN-T led to a dose-dependent decrease in several systemic inflammatory markers. By day 7, the termination of the experiment, levels of TNFα, and IL-6 were decreased in a dose-dependent manner. This reduction correlates with the protection from bacterial dissemination and effective control of the inflammatory response (Figure 7). Because of the immediate mortality of all animals on day 1 in the untreated group, no values are available for this biomarker.

### 2.5. Tolerability Including Histopathology–Rat Periodontitis Model

The potential side effects of the formulation on periodontal tissues in Wistar rats were evaluated by microinjections into the tissue. Based on the dosage of 20 mg/kg bodyweight, this corresponds to a 70–100-fold higher dosage of a later recommended human dosage.

Over a 10-day observation period after microinjecting MIN-T, physiological parameters such as body weight, food intake, and water consumption remained stable, indicating no pathological effects from the application. Additionally, there were no observable behavioural changes in the rats, such as altered body posture, decreased physical activity, changes in fur, or behaviours indicative of pain that would suggest a negative impact from the formulation.

**Figure 8:**
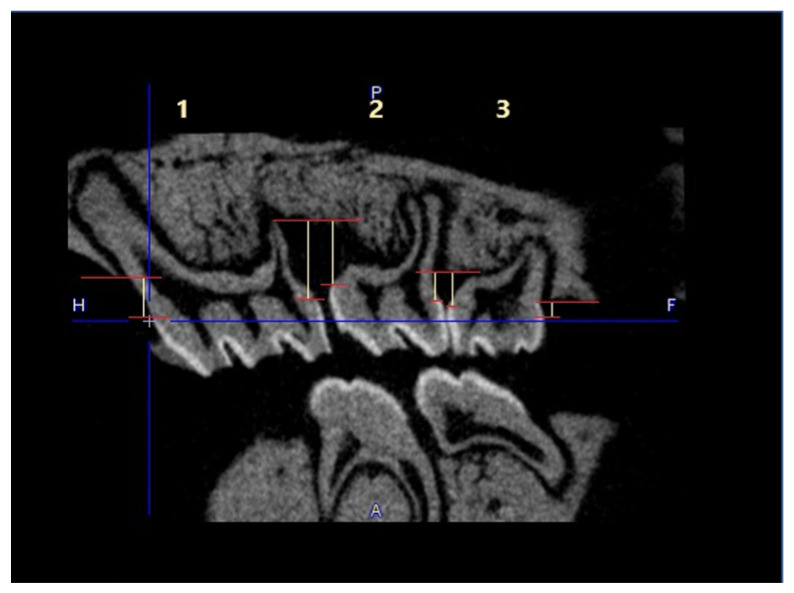
Overview of the measured distances used for the statistical calculations. The three upper molars are marked with 1, 2, and 3. The distances are marked as yellow lines in red borders. This example is taken from the *P. gingivalis* infected, but not treated group (PG).

No visible changes were noted in the periodontium. Furthermore, CT scans revealed no alterations in the jawbone area. Due to technical constraints, the experiment was confined to the upper jaw, and alveolar bone loss was quantified using micro-computed tomography (μCT). Measurements were taken from the cemento–enamel junction (CEJ) to the alveolar bone crest (ABC). For each analyzed rat (23 in total), 12 measurements were conducted: three times per molar at two sites (left and right), doubled for statistical replication. The specific distances utilized for statistical analysis are detailed in Figure 8.

**Figure 9:**
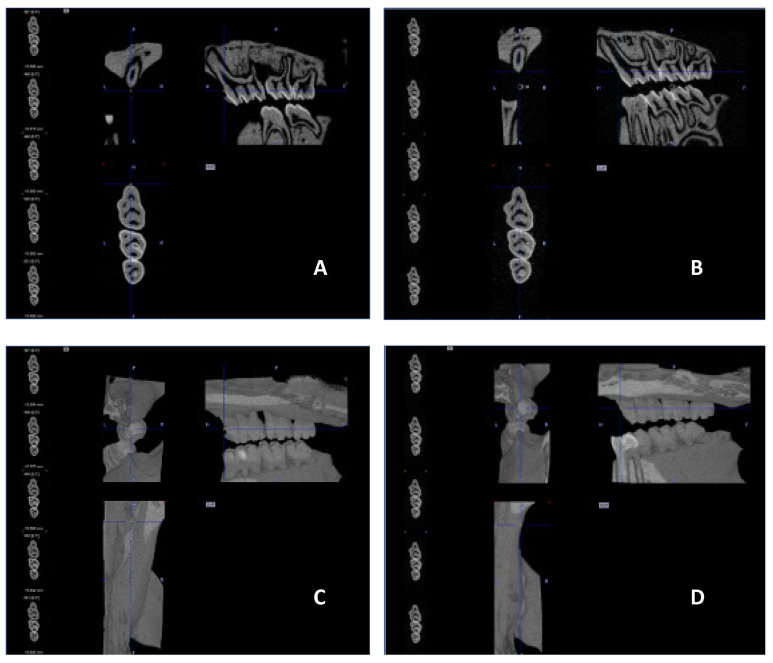
µCT images showing the treatment effect of the novel formulation against a *P. gingivalis* infection in the rat ligature model. (**A**,**B**) sagittal section of the head/jaw axis at the level of the bone/teeth (**C**,**D**) sagittal section of the head/jaw axis (the same scan) after adding bone and tissue mass (**A**,**C**) infected and untreated animals (PG), (**B**,**D**) infected and treated animals (PG+MIN-T).

Figure 9 presents a representative CT analysis of bone loss in *P. gingivalis*-infected animals (both minocycline-treated and controls. The beneficial effect of the treatment is clearly visible with regard to bone loss. Comparison of the CEJ-ABC distance shows a significant bone loss in the non-treated animal (A + C, PG), which was remarkably diminished by the treatment (B + D, PG+MIN-T).

**Figure 10:**
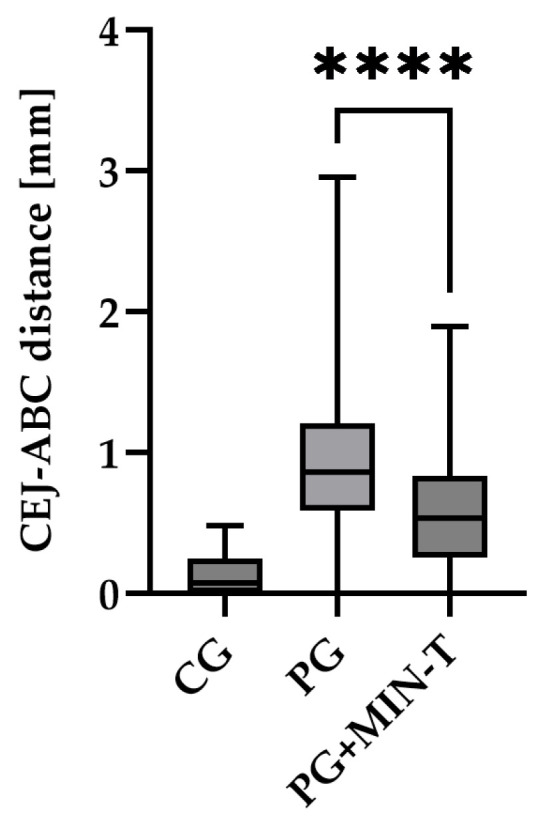
CEJ-ABC distance (mm) measured as compared to the control scan executed before the start of the experiment. Treatment with MIN-T showed significant improvement in bone loss throughout the experiment. CG—control group; PG—non-treated group; PG+MIN-T—treated group. Statistical significance was calculated via one-way ANOVA. CEJ-ABC, cemento–enamel junction-alveolar bone crest. **** *p* < 0.0001.

This observation has been statistically validated, as shown in Figure 10. Aggregated measurements across all evaluated molars (1st, 2nd, and 3rd upper molars) indicate a significant reduction in bone loss in the treated group PG+MIN-T, with a *p*-value of <0.0001 compared to the non-treated group (PG). This demonstrates the antibiotic’s effectiveness over 31 days following treatment with the novel formulation.

**Figure 11:**
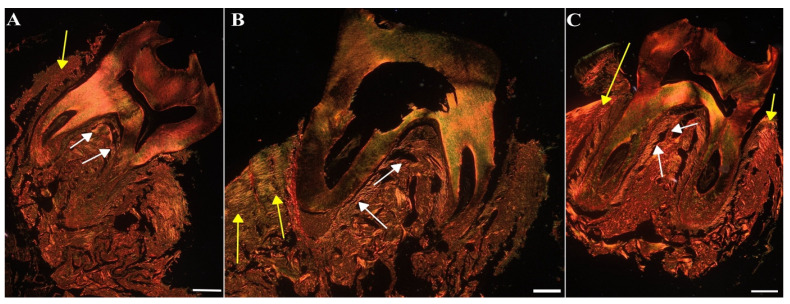
Representative images of the jawbone of Wistar rats: Sirius red staining in picric acid used for the determination of different collagen types. (**A**) Control group (CG), (**B**) periodontitis group (PG), (**C**): periodontitis + MIN-T group (PG+MIN-T). Scale = 200 µm. Yellow arrows mark compact bone, white arrows mark trabecular bone.

To assess the impact of MIN-T on the bone structure changes during periodontitis, immunohistochemical analysis of rat jawbone was conducted. Picrosirius red (PSR) staining was used to distinguish young and maturated collagen fibres in both compact and trabecular bone (indicated by yellow and white arrows, respectively, in Figure 11).

**Figure 12:**
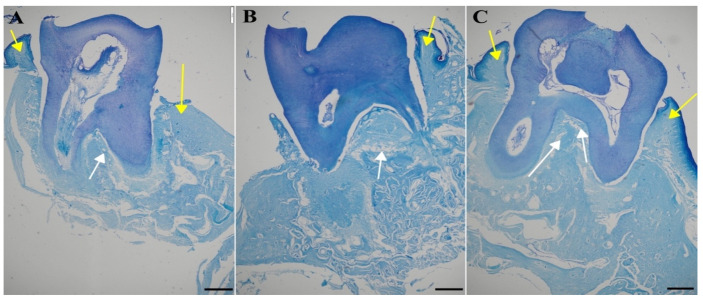
Representative images of the jawbone of Wistar rats: toluidine blue staining used for the measurement of the microarchitecture of the trabecular bone. (**A**) Control group (CG), (**B**) periodontitis group (PG), (**C**): periodontitis + MIN-T group (PG+MIN-T). Scale = 200 µm. Yellow arrows mark compact bone, white arrows mark trabecular bone.

Another staining using toluidine blue was used to assess the microarchitecture of the trabecular bone and calculate the BV/TV parameter, which quantifies trabecular bone volume. Again, in the figure the compact and trabecular bone are clearly visible (indicated by yellow and white arrows, respectively, in Figure 12).

Significant differences (*p* < 0.05) were observed between the groups (Table S2): control (CG), untreated periodontitis (PG), and periodontitis pretreated with MIN-T (PG+MIN-T). Post-treatment, the trabecular bone volume (BV/TV) was slightly reduced in the treated group (PG+MIN-T), while it remained similar in both the control (CG) and untreated periodontitis (PG) groups. Additionally, there was a significant (Table S2) increase in trabecular separation (Tb.Sp) in the maxillary bone of the treated group (PG+MIN-T) compared to the control (CG) and untreated (PG) groups. This increase may indicate a potential weakening of the trabecular bone’s microarchitecture in the individuals who received the drug. Other parameters, such as trabecular number (Tb.N) and trabecular thickness (Tb.Th), showed no treatment effects on the microarchitecture of the trabecular bone (Table S2). For the trabecular bone, coarse collagen bundles were more prevalent in both the control group (CG) and the treated group (PG+MIN-T) compared to the infected, untreated group (PG) (Table S3), where bone tissue structure appeared significantly weakened. Because the Tb.Sp parameter is only one out of several indicators describing the microarchitecture of the trabecular bone and other parameters like Tb.Th and Tb.N are not affected, a significant deterioration in bone structure can be denied at this stage of investigation.

The ratio of fine-fibrous young to coarse-fibrous maturated collagen (Y/M_compact_) in the maxillary compact bone was significantly (*p* < 0.05) lower in the treatment group (PG+MIN-T) than in both the control (CG) and untreated (PG) groups (Table S3). This suggests that the local administration of the minocycline formulation may have induced the synthesis of new collagen. Furthermore, the proportion of maturated coarse-fibrous collagen (%M_compact_) in the compact bone of the maxilla was higher in both the treated (PG+MIN-T) and untreated (PG) groups compared to the control group (CG) (Table S3), indicating a compromised bone structure in the area due to the infection with *P. gingivalis*.

As for the efficacy model, the systemic burden of minocycline after microinjection into the animals was measured by an adapted LC-MS/MS method. The further optimization of the method for the rat serum as used biological matrix resulted in an LLOQ of 5 ng/mL minocycline. Using the mentioned LLOQ, it was not possible to quantify minocycline after the treatment of 20 mg/kg in the serum of any of the investigated animals. Therefore, no data can be presented here. To illustrate this, Figure S2 shows a representative HPLC chromatogram of one sample, compared with a chromatogram of a reference run. Clearly, in the sample the signal for minocycline at 2.12 min is far below any possibility for quantification.

## 3. Discussion

The aim of this study was the non-clinical investigation of a novel adjunctive approach for the local placement of an antibiotic in periodontitis therapy. Thereby, a locally applied antibiotic has several advantages compared with systemic application, mainly because of drastically reduced side effects and less generation of resistance [18]. Minocycline as an active pharmaceutical ingredient has been used in different formulations for decades as an adjuvant for therapy [32,33]. The use of such a well-known active substance with the intended dosage and indication has the advantage of giving certainty concerning efficacy and low to non-toxic effects [23,24,25]. Nevertheless, a novel formulation was developed to add certain advantages to a new product. It releases the antibiotic over several weeks in the periodontal pocket. This need for a bacterial-free environment is reflected by the finding of a recolonization of the periodontal pockets within 60 days after SRP alone [34] and a higher efficacy of chlorhexidine chips compared to chlorhexidine gels as adjunctive therapy in periodontitis, where the chips have a prolonged release of the antiseptic compared with the gels [35,36]. A prolonged time frame with no bacterial reinfection enables wound healing and the reduction of local inflammation for a prolonged period. The later effect is supported by the immunomodulating activity of minocycline, which is clearly an add-on effect compared to other anti-infectives, like chlorhexidine [37,38,39,40]. Of course, locally applied antibiotics are still antibiotics with all pros and cons, e.g., a possible development of resistances. For that reason, the usage of such compounds should always be carefully considered according to a proper benefit. Other disadvantages of such a solution might be reaching a sufficient concentration of the antimicrobial at the respective site due to difficulties placing the drug properly and a continuous crevicular flow. It might be challenging for dental professionals to place the drug, in part because special equipment is needed, and even still the drugs are often expensive [41].

Although no relevant toxic events are assumed for the active ingredient, the novel formulation must be examined for its proof-of-concept, as well as for a possible systemic burden of minocycline after a local application. In that regard, toxicity that may be related to the excipients used must be investigated [42]. In addition to this, the status of possible resistance of relevant oral pathogens against minocycline is also of high interest. Because of its long clinical use, the probability of existing resistance against minocycline is high [43,44,45]. Finally, the behaviour of the formulation after placement into periodontal pockets is of high interest. From a material as well as a pharmaceutical point of view, it is important to understand how exactly the formulation disappears in the pocket.

In general, the presented study was able to find answers to most of the aforementioned questions. First, MIN-T was successfully tested in two different animal models, both of which are well-established in the scientific community for the investigation of periodontitis. The chamber mouse model demonstrated a dose-dependent effect on survival rates. Due to technical constraints, the formulation had to be placed into the chamber before its implantation. Of course, this does not reflect any relevant clinical situation where the drug is applied after a detected infection. On the other hand, such an approach has the advantage of showing a controlled release over the entire duration of the experiment. The positive outcomes in treated animals indicate the formulation’s capability for sustained release of minocycline over several weeks. According to the protocol, *P. gingivalis* inoculation occurred 10 days post-implantation, during which the formulation was already present in the newly formed connective tissue within a serum-rich environment. Under these conditions, minocycline is known to convert into its less microbiologically active epimer, 4-epiminocycline, and undergo further degradation [46,47,48]. This is suppressed, as already described in the former in vitro studies [22,27] in which the developed innovative complex of minocycline with magnesium stearate stabilizes the active ingredient, finally enabling a microbiological activity throughout the whole 17-day experiment. The significant systemic reduction in key inflammatory markers (IL-6 and TNFα) highlights clearly the impact of successfully treating a local infection and corresponds well with findings in humans, where periodontitis-affected patients have higher levels of inflammation markers compared with periodontal healthy people [49,50,51,52]. Furthermore, even the highest dose of the locally administered antibiotic formulation (80 mg/kg) did not result in significant systemic levels of the active molecule, thereby protecting the local and systemic microbiomes from damage and potential development of antibiotic resistance.

Although the chamber model is widely used to investigate and evaluate treatments targeting *P. gingivalis* [53,54], in general, the rat periodontitis model more accurately replicates periodontal pathology, such as tissue degradation and bone loss, despite its limitations [55,56]. One such limitation involves, again, the suboptimal placement of the MIN-T formulation. Again, technical issues were responsible for an experimental regime, which does not reflect a clinical situation at all. Because no applicable periodontal pockets can be formed in the animal model which corresponds to the given size and dimensions of the new MIN-T formulation, the threads must be grounded and injected once at six distinctive places directly into the periodontal tissue. This grinding significantly affects the dissolution and release rate of the antibiotic due to the increased surface area of the resulting particles compared to the original compact form. In addition, an injection of MIN-T into the small space of the gingival tissue always entails the risk of minor injuries. Therefore, an infection before the application of MIN-T (e.g., oral gavage model) would most likely lead to an invasion of the bacteria due to the previously occurred micro-injuries into the periodontal tissues or even blood vessels. Such a procedure could contribute to the development of an undesirable inflammatory state. Despite these less-than-ideal circumstances, the model still demonstrated the formulation’s applicability. The stabilization of the antibiotic by magnesium stearate embedded in the MIN-T formulation maintained a sufficient compound level for an effective antibiotic response over the whole 32 days, even though the animals were challenged with freshly prepared *P. gingivalis* suspension every second day after the application. In addition, minocycline was released from the tissue sufficiently. Interestingly, in the control group no toxic effect was detected from the microinjection itself. Thereby, MIN-T had a remarkable effect on reducing bone loss compared to the untreated periodontitis group (PG), with statistical significance at the *p* < 0.0001 level.

On the other hand, the histopathological results present some complexities. In both humans and animals, bones are classified by shape into categories such as long (e.g., tibia), short (e.g., fingers), irregular (e.g., vertebrae), and flat (e.g., skull) [57]. Additionally, bone tissues are histologically distinguished into two types: compact and spongy, which differ in structure and metabolic activity [57]. Notably, remodeling in compact bone is less dynamic than in trabecular (spongy) bone. Pathological conditions typically lead to a reduction in the bone volume fraction (bone volume/tissue volume, BV/TV), characterized by a thinning of the bone structure, as well as the deterioration of other microstructural parameters such as trabecular thinning, increased trabecular separation, and a decrease in the trabecular number [57].

The present study shows that the bone volume fraction BV/TV in the maxilla of the treated animal group (PG+MIN-T) was significantly reduced in both infected groups (PG and PG-MIN-T) compared to the control group (CG). This clearly indicates the above-mentioned typical reduction of that value after an infection with *P. gingivalis*, whereas a treatment with MIN-T was not able to inhibit the loss of the tissue. This is in contrast with other results of the study. As mentioned, the µCT clearly indicates a lower total bone loss by showing a significantly reduced CEJ-ABC distance (mm) in the PG+MIN-T group compared to the PG group. Whereas these results are statistically relevant, the difference between the PG group and the CG group is much higher compared to the PG and PG+MIN-T group. This fits clearly into the picture that an infection with *P. gingivalis* always affects the jawbone of the animals regardless of the application of an antibiotic or not. The treatment can only minimize the effect but not fully block bone loss on the organ (µCT) as well as on the tissue (BV/TV-values) level. In addition, no differences were observed in trabecular thickness or trabecular number in the maxilla between the two disease-affected groups, PG and PG+MIN-T.

Interestingly, in the PG+MIN-T group, the size of the intertrabecular spaces in the maxillary bone was significantly larger, compared to the control (CG) and non-treated (PG) groups. This may suggest a potential weakening of the trabecular bone microarchitecture as a first impression. The trabecular space (Tb.Sp) parameter is thereby only one of several indicators describing the microarchitecture of the trabecular bone. If an increase in Tb.Sp would be associated with a thinning of the beads (a decrease in trabecular thickness, Tb.Th) and a decrease in the number of bone beads (Tb.N.), then this would indicate a significant deterioration in bone structure. The study results clearly show that MIN-T is not affecting other parameters, such as Tb.N and Tb.Th. Following this information, the application of MIN-T seems not to have a significant negative influence on the microarchitecture of the trabecular bone. For a complete picture, bone strength tests will be a valuable source of information. Therefore, it is planned to expand the analyses to include such techniques in future studies. Additionally, the non-treated periodontitis group PG exhibited a decrease in the percentage of maturated coarse-fibrous collagen and a significant increase in the percentage of fine-fibrous collagen as well as the ratio of young fine-fibrous to maturated coarse-fibrous collagen bundles within the trabecular bone of the maxilla, compared to both control and treated animals PG+MIN-T. This might indicate disturbances in the collagen network of the trabecular bone in the jaws of infected rats, suggesting that drug administration positively affects bone turnover by eradicating bacteria, and indicating induction of new collagen synthesis following an anti-infective treatment.

Moreover, the percentage of coarse-fibrous collagen (%M_compact_) in the compact bone of the maxilla was significantly increased in both untreated (PG) and treated (PG+MIN-T) animals compared to controls (CG), signaling a compromised bone structure in this area of the maxilla due to an infection with *P. gingivalis*. In summary, while the administration of MIN-T does not enhance the microarchitecture of the trabecular bone in the maxilla, it also does not significantly deteriorate it. Furthermore, drug administration does not significantly impact collagen synthesis, which could potentially increase the stability of collagen fibres and improve the condition of the trabecular bone.

Again, the systemic burden of animals with the antibiotic was investigated. At a dosage of 20 mg/kg, no minocycline was detected in the systemic circulation. So, all in all, it can be assumed that in humans the systemic burden will be neglectable in that regard, too. This is in accordance with reports for comparable products. A 15% doxycycline containing in situ forming gel was applied in a phase I clinical study in dental pockets. Besides high initial local concentrations in GCF and saliva, 19 out of 20 patients were tested negative at all time points for serum levels of the tetracycline derivative [58].

Furthermore, in an additional experiment, it could be shown that the current susceptibility of relevant human oral pathogens against minocycline is not diminished. The determined MICs are in agreement with recently published values [44] and did not change during the passages, with one exception. In one *F. nucleatum* strain a clear increase of four steps in susceptibility was observed. However, the values were still very low, and the effect was stable through all following passages and did not increase. Therefore, that strain would also not count as resistant, and the efficacy of MIN-T is not affected due to a sufficient minocycline concentration above the MIC.

Finally, it was possible to demonstrate the degradation and disappearance behaviour of the thread after application. First, the MIN-T swells significantly in a very few hours and keeps that volume for several days. This might enhance the residence feature of the threads because the higher volume might lead to a better filling of and therefore an enhanced adhesion in the periodontal pocket. This behaviour can be explained by the penetration of water into the formulation, due to the polar polyethylene glycol (PEG) groups. This penetration is also clearly reflected by the formation of pores visible at the surface of the threads. The resulting more aqueous surroundings lead to the start of the hydrolytic degradation of the poly(lactic/glycolic)-PEG block-polymer. It is known that degradation and release processes from PLGA polymers are complex [59]. The released monomers are alpha-hydroxy acids, which might cause very low pH values and autocatalytic polymer degradation [60]. It has been shown that polymer degradation of PLGA-PEG polymers compared to PLGA polymers starts earlier due to the initial higher water penetration, but is ultimately slower because autocatalysis can be avoided [61]. In addition, the minocycline-lipid-complex shields minocycline against the aqueous phase, resulting in a very slow release of the active ingredient and also stabilizes the drug against degradation in an aqueous environment. Again, the acidic conditions are responsible for the degradation of the MLC itself. All mentioned processes led to a time-controlled degradation of the formulation. This degradation is reflected by the stepwise disaggregation of the solid thread to particles of different sizes on day 38, whereas following the degradation itself, it was not possible due to technical reasons. In the end, due to the increased formation of water-soluble degradation products, polymer will more rapidly disappear and the clearance from the periodontal pocket will be enhanced. This results in a short time with concentrations of minocycline below the needed MIC. The presented data nicely illustrate the findings of Schmid et al. [22], who showed antimicrobial activity of MIN-T for the duration of 42 days in vitro by simulating an SCF-flow. In the end, only the planned clinical studies will show the real behaviour in humans.

## 4. Materials and Methods

### 4.1. Generation of the Threads

The pharmaceutical development, manufacturing, and characterization of the PEG-PLGA and minocycline lipid complex (P-MLC) extrudates have been described in previous publications [27,28]. In short, minocycline (Ontario Chemicals Inc., Guelph, ON, Canada) was chelated with magnesium stearate (Magnesia GmbH, Lüneburg, Germany) in a molar ratio of 1:2. Subsequently, the complex was mixed with the desired PEG-PLGA_6P_ polymer (Seqens SAS, Aramon, France) and cryo-milled. The used PEG-PLGA thereby contains a lactide/glycolide ratio of 50%/50% with an overall molweight of around 40 kDa and ca. 11% of substituted PEG as an end group of the PLGA co-block-polymer. This composition was utilized for the hot melt extrusion with a 600 μm device (ThreeTec, Seon, Switzerland). The extrudates contained 11.5% (*m*/*m*) of minocycline. All minocycline formulations were also kept thoroughly in the dark throughout the experiments.

### 4.2. In Vitro Swelling and Degradation Studies

The swelling behaviour and degradation of the formulation were investigated on the extrudates. For the latter experiments, the extrudates were stored in PBS (pH 6.8, 4 mL) without light exposure at 37 °C for up to 38 days under slight shaking (80 rpm). The extrudates were observed by a laser scanning microscope (LSM) or a scanning electron microscope (SEM) before and after different exposure times.

The swelling behaviour of the threads was documented by a laser scanning microscope (VK-1000/1050, Keyence Germany GmbH, Neu-Isenburg, Germany) before experimentation, and after 2, 3, 4, 7, 9 and 11 days, respectively. These time points were chosen according to pre-studies. After day 4, no further swelling was observed, and the experiment was finished on day 11. Samples were taken out at each time point from the PBS buffer, immediately investigated by LSM, and placed back into the PBS buffer. The volume increase of the thread was calculated by image evaluation software (Multifile Analyzer, Keyence, Germany GmbH).

Further, the surface morphology of the extrudates was documented before experimentation, after 3 h, and then after 3, 22, and 38 days, respectively, by SEM (Quanta 3D FEG from FEI company). One sample was taken out at each time point from the PBS buffer and immediately prepared for investigation using SEM analysis. Samples were prepared using Nanosuit technology (NanoSuit^®^ from NanoSuit Inc., Hamamtsu, Japan) to obtain a conductive layer on the surface. The following magnifications were used: 200× and 10,000×.

### 4.3. Determination of Resistance

A potential development of resistance against minocycline (minocycline hydrochloride) was studied in several relevant oral strains. In all 11 clinical isolates (three *P. gingivalis*, four *F. nucleatum*, and four oral *streptococci*) were included.

The cultivation of a subgingival biofilm and isolation of respective bacterial strains was approved by the Ethical Committee of the Canton Bern (KEK 096/15). Only samples from individuals who did not receive antibiotic treatment 2 months before the date of collection were included. Identity was confirmed by PCR using species-specific primers. Bacterial strains were kept frozen at −80 °C. About one week before experiments, they were sub-cultured and passaged 2–3 times on tryptic-soy-agar plates with 5% sheep blood.

The method of inducing resistance was adapted to the previously described procedures [29,30,31]. In short, the strains were passaged on Wilkins–Chalgren agar plates (Oxoid) with subinhibitory MIC concentrations (about ^1^/_4_–^1^/_8_ MIC) of minocycline up to 50 passages. Before and after every 10 passages, MICs were determined by using the microdilution technique.

### 4.4. Animal Models

All animal procedures were reviewed and approved by the 1st Regional Ethics Committee on Animal Experimentation, Kraków, Poland (approval number: 167/2021) and carried out in rooms with high efficiency particle accumulation-filtered air within the animal facility at the Jagiellonian University (Krakow, Poland). Specific pathogen-free (SPF) female BALB/c mice (8–12 weeks old) and female Wistar rats (8 weeks old) were purchased from Janvier Labs (France). All animals were housed in individually ventilated cages; the average temperature in animal rooms and treatment room was: 22 ± 2 °C; average humidity in animal rooms and treatment room was: 55 ± 10%; light cycle consisted of: 12 h of day and 12 h of night. All animals were fed a standard laboratory diet and allowed water ad libitum. The health status of animals was monitored in accordance with the Federation for Laboratory Animal Science Associations guidelines.

Bacteria used for the infection in the animal models were handled as followed: *P. gingivalis* wild-type (W83; ATCC, Rockville, MD, USA) were plated on tryptic soy broth (TSB) agar plates (5% sheep blood, supplemented with l-cysteine (0.5 mg/mL), hemin (5 μg/mL), and vitamin K (0.5 μg/mL)) and grown under anaerobic conditions at 37 °C for 7 days. Two days before infection, bacteria were inoculated in TSB with hemin, vitamin K, and l-cysteine and grown (1st day–preculture, 2nd day–culture) until a mid-log phase OD_600_ of 0.6. On the day of infection, bacteria were washed twice in sterile PBS and prepared at a final concentration of 2 × 10^8^ bacteria/0.1 mL in sterile PBS/mice (subcutaneous chamber model) or 1 × 10^10^ bacteria/1 mL in sterile PBS + 2% methylcellulose/rat (periodontitis/oral gavage model) and used immediately for infection. Before each infection, bacteria were prepared from a fresh bacteria culture.

### 4.5. Mice Subcutaneous Chamber Model

Specific pathogen-free (SPF) 8–12-week-old BALB/c were purchased from Janvier Labs (Le Genest-Saint-Isle, France). In line with an established protocol [62], titanium chambers were surgically implanted subcutaneously into the backs of the animals (dorsolumbar region, 42 mice in total), after placing MIN-T in six different dosages (0 (control group), 1, 10, 25, 40, 80 mg/kg body weight/7 animals per group) into the chambers. After the complete healing of the incisions (10 days) and the interior encapsulation of the coil by a thin vascularized layer of fibrous connective tissue, 0.1 mL suspensions of *P. gingivalis* were injected into the lumen of the chamber. Based on previous tests, a lethal dosage containing 2 × 10^8^ of *P. gingivalis* from an overnight culture in 100 μL of PBS was chosen. Chamber fluids (10 μL) were aspirated using a hypodermic needle (25G) at 24, 72, 120, and 168 h intervals and analyzed for the presence and levels of minocycline by an already established HPLC-based method. *P. gingivalis* CFUs were measured to determine the viability of the pathogens. All surviving animals were sacrificed on day 7 and the blood was collected from the venous sinus for the evaluation of minocycline systemic levels and inflammatory markers (Figure S3).

### 4.6. Rat Periodontitis Model

Specific pathogen-free (SPF) 8 week old female Wistar rats purchased from Janvier Labs (Le Genest-Saint-Isle, France) were used in the experiment (ten per group for the *P. gingivalis* challenge only (PG) and *P. gingivalis* + MIN-T (PG+MIN-T) and three per group for MIN-T only (CG)). In line with an existing scientific protocol [63], the animals were pre-treated with an antibiotic mix containing sulfamethoxazole (870 μg/mL) and trimethoprim (170 μg/mL) for 8 days. After this, in two groups (CG, PG+MIN-T), the MIN-T formulation was provided. Because of the dimensions of the threads, the formulation needed to be ground for the application. Then, a total of six microinjections (35 μL per site-inner outer with 30G cannulas) in the 1st, 2nd, and 3rd molar of the upper jaw were done, two for each tooth. This resulted in a final dosage of 20 mg/kg. Thereby, the experiment was limited to the upper jaw due to technical limitations. After this, animals from the PG and PG+MIN-T groups were challenged a total of six times with 1 × 10^10^ CFU each time every second day (for a total of 12 days for the challenge).

After 32 days the animals were sacrificed, and the alveolar bone loss was measured by micro-computed tomography (μCT) as the distances from the cemento–enamel junction (CEJ) to the alveolar bone crest (ABC). In addition, the blood of the animals was collected to determine the systemic burden of minocycline after 32 days (Figure S4).

#### 4.6.1. Micro-Computed Tomography Analysis

To determine bone loss High Resolution Animal Computed Tomography (Micro-CT, MILabs, The Netherlands) was used. All animals were scanned at two time points (before treatment (T_0_) and on the day of termination of the experiment (T_END_)). Imaging was performed at an ultra-focus magnification, 50 kV source voltage, and 0.21 mA current. Three-dimensional images were obtained using the PMODE software (vers. 4.3; Fällanden, Switzerland). To assess the alveolar bone loss, a linear distance from CEJ to ABC of each tooth of the lower and upper jaw was measured. Each measurement was performed three times, and the data are presented as the mean ± standard deviation (SD). The results are presented as the distance after subtracting the basal measurement (T_0_) from the measurement obtained at the endpoint of the procedure (T_END_).

#### 4.6.2. Bone Density

Maxillary bones from every animal were isolated, soft tissue was removed and decalcification in an Osteomall commercial decalcifier (Sigma-Aldrich, St. Louis, MO, USA) was performed. Decalcified samples were dehydrated in graded ethanol solutions and embedded in paraffin. From each rat, coronal (frontal) sections (5 µm thickness) from the first molar, second molar, and third molar region were cut with a microtome. For the trabecular bone, Toluidine blue staining was performed. To differentiate collagen type in trabecular and compact bone picrosirius red (PSR) staining was employed. The sections were analyzed with an Olympus CX43 microscope (Olympus, Tokyo, Japan) equipped with filters providing circularly polarized illumination. Objective magnification of 4× and 10× was used to collect images. The bone microarchitecture was assessed using ImageJ software (NIH, Bethesda, MD, USA). The following parameters were determined: bone volume (BV/TV), mean trabecular thickness (Tb.Th mean), maximal trabecular thickness (Tb.Th max), mean trabecular space (Tb.Sp mean), maximal trabecular space (Tb.Sp max), and trabecular number (Tb.N), as well as the distribution of thin (immature) collagen fibres, the distribution of thick (mature) collagen fibres, and the proportion of the mature and immature collagen fibres in the trabecular and the compact bone. The analysis was done about all molars, and corresponding values are presented in Tables S2 and S3. For these histological analyses, ImageJ software (NIH, Bethesda, MD, USA) was used, according to the literature [64].

### 4.7. Analytical Methods

#### 4.7.1. qPCR Analysis of *P. gingivalis* in Mice Chamber Fluids

DNA was extracted from chamber fluid using the DNeasy Blood & Tissue Kit (Qiagen, Hilden, Germany) according to the manufacturer’s protocol. TaqMan qPCR was performed with Kapa Probe fast qPCR Mix (Rox Low) on a Bio-Rad CFX96 Real-Time System C1000 Touch ThermalCycler with the forward (5′-AGCAACCAGCTACCGTTTAT-3′) and reverse (5′-GTACCTGTCGGTTTACCATCTT-3′) primers and 6-FAM-TACCATGTTTCGCAGAAGCCCTGA-TAMRA as the detection probe. The primers were based on a single copy of *P. gingivalis* arginine-specific cysteine-proteinase gene. The samples were run in duplicate in a total volume of 10 μL, containing 100 ng of DNA. TaqMan Universal PCR Master Mix (2×) (Kapa Biosystems, Wilmington, MA, USA), and the specific set of primers (final concentration, 5 μM) and probe (final concentration, 4 μM) (GenoMed S.A., Warszawa, Poland), corresponding to 562.5 nM of forward and reverse primers and 100 nM of the probe. After an initial incubation step of 2 min at 50 °C and denaturation at 95 °C for 20 s, 40 PCR cycles (95 °C for 20 s and 60 °C for 30 s) were performed. The number of copies of the *P. gingivalis* genome was calculated by matching Cq values with a standard curve prepared from serial dilutions of cultured *P. gingivalis* W83 (WT).

#### 4.7.2. Chamber Fluid Minocycline Concentration (Mice)

To 4 μL of chamber fluid, phase A (0.1% TFA in distilled water) was added to receive a final volume of 65 μL. Proteins were precipitated in the presence of 13% TCA on ice for 30 min, and samples were centrifuged for 15 min (13,000 g) at 4 °C. The supernatant was collected and 50 μL of sample was injected onto the column (Phenomenex, Aeris 3.6 μm Peptide XB-C18, LC 150 mm × 4.6 mm) and separated by using a Shimadzu NexeraX2 system. Post injection, the column was rinsed with 5% phase B (80% acetonitrile, 0.08% TFA) for two column volumes and then minocycline was eluted in a rising gradient of phase B (5–30% in 15 min at 1.5 mL/min) for each sample. Minocycline was detected at 355 nm. After each run, the column was rinsed with 100% phase B for 5 min at 1.5 mL/min and then equilibrated for another 5 min with 5% phase B at 1.5 mL/min. The minocycline amount in the sample was calculated based on a standard curve (AUC).

#### 4.7.3. Serum Cytokine Measurement (Mice)

Concentrations of IL6 and TNFα in serum were measured using a commercially available kit (Milliplex MAP Mouse High Sensitivity T-cell Magnetic Bead Panel cat. MHSTCMAG-70pk, Sigma-Aldrich (Merck, Darmstadt, Germany)) according to the manufacturer’s protocol. In short, standards, internal controls, and samples were pipetted into the wells. Beads suspended in the assay buffer were added and the plate was incubated overnight at 4 °C on an orbital shaker. Then, the plate was placed on a magnet and beads were washed three times with the buffer before the detection antibodies were added and the plate was incubated for another hour at room temperature on a shaker. Streptavidine-phycoerythrin was added and samples were incubated for a further 30 min at room temperature. Beads were washed three times, suspended in Drive Fluid PLUS, and the signal was measured on a MAGPIX instrument (Luminex xMAP, Bio-Rad Laboratories GmbH, Feldkirchen, Germany) using xPONENT software (ver. 4.1).

#### 4.7.4. Serum Concentration of Minocycline (Mouse and Rat)

As an analytical standard minocycline-HCl was obtained from Sigma-Aldrich (Merck KGaA, Darmstadt, Germany) as a pharmaceutical secondary standard. The internal standard Minocycline-D7 was purchased from TRC (Toronto, ON, Canada).

Minocycline itself was quantified by LC-MS/MS by using calibration curves and normalization related to the deuterated internal standard minocycline-D7.

The working solutions for the calibration standards were prepared by diluting minocycline with ACN/H_2_O (90/10; *v*/*v*). The concentration of minocycline in the working solutions covered a range of 5 ng/mL to 1000 ng/mL (rat serum) and 20 ng/mL to 100 ng/mL (mouse serum), respectively. The working solution of the internal standard was prepared by diluting minocycline-D_7_ with ACN/H_2_O (90/10; *v*/*v*) to a final concentration of 10 ng/mL.

The preparation of the study samples was based on protein precipitation. Briefly, 60 µL of the internal standard working solution was spiked with 20 µL of ACN/H_2_O (90/10; *v*/*v*) and 20 µL of the serum sample, followed by incubation at 5 °C for 10 min. The samples were vortexed, centrifuged and the supernatant was further processed by using centrifugal filter devices. Finally, 30 µL of the filtrate was diluted with 60 µL water and transferred into HPLC vials. Calibration samples were prepared by spiking 60 µL of the internal standard working solution with 20 µL of the calibration working solution and 20 µL blank matrix. Blanks were prepared by spiking 60 µL of the internal standard working solution with 20 µL of ACN/H_2_O (90/10; *v*/*v*) and 20 µL blank matrix. Double blanks were prepared by spiking 60 µL of ACN/H_2_O (90/10; *v*/*v*) with 20 µL of ACN/H_2_O (90/10; *v*/*v*) and 20 µL blank matrix. Calibration samples, blanks, and double blanks were processed as described for the study samples.

The quantification of minocycline was based on its separation by UPLC (1290 Infinity II, Agilent Technologies, Waldbronn, Germany) on a C_18_ stationary phase (Acquity UPLC CSH C18 with 1.7 µm, 130 Å, 100 × 2.1 mm, Waters, Eschborn, Germany) at 30 °C using a gradient elution with a mobile phase system consisting of 5 mM ammonium acetate in H_2_O (pH 2.7) and 5 mM ammonium acetate in H_2_O/MeOH (5/95; *v*/*v*). The mass spectrometry analysis was carried out on a hybrid triple quadrupole/linear ion trap mass spectrometer (QTRAP 5500+, AB Sciex Germany GmbH, Darmstadt, Germany) using electrospray ionization (ESI) and multiple reaction monitoring (MRM). The MRM transitions are summarized below (Table 2).

Data acquisition and processing was carried out using Analyst 1.7.2 (SCIEX), SCIEX OS 2.1 (SCIEX) and Excel 16.0 (Microsoft Corp., Redmond, WA, USA). The concentration of the analytes was calculated by applying the internal standardization method. The calculation of the minocycline quantities was based on the area ratio of the analyte to the internal standard plotted against the respective concentration ratios of the calibrants. Data points were fitted with a weighting factor of 1/×2 using linear regression with the method of least squares.

## 5. Conclusions

This study presents key findings from the non-clinical development of a locally applied minocycline-containing pharmaceutical formulation. In vitro studies had previously demonstrated prolonged activity, a result that was confirmed in widely accepted animal models for the disease within this study. Furthermore, the potential systemic burden of the antibiotic was investigated and found to be below levels of concern. An additional important aspect of this research was the resistance profile of oral pathogens to the used antibiotic, which showed that clinical isolates remain susceptible to this long-established antibiotic.

Given that minocycline has been known and used safely in similar applications for decades, a relatively rapid processing through clinical development is anticipated.

## Figures and Tables

**Table 1:** MIC values of minocycline determined against several clinical isolates of representive oral pathogens. Each MIC value was measured after the amount of the corresponding number of passages, whereas during all bacterial cultivation experiments minocycline was present at ^1^/_4_ to ^1^/_8_ of the MIC, measured at passage 0.

Strain	MIC [µg/mL] After Passages					
	0	10	20	30	40	50
*S. gordonii* BeTa9-2	0.063	0.063	0.063	0.063	0.063	0.063
*S. constellatus* BeTa7-1	0.031	0.031	0.031	0.031	0.031	0.031
*S. mitis* BeTa7-2	0.125	0.125	0.063	0.125	0.063	0.063
*S. oralis* JM933	0.031	0.031	0.031	0.031	0.031	0.031
*P. gingivalis* TR60219	0.016	0.016	0.016	0.016	0.016	0.016
*P. gingivalis* BeOR6-1	0.016	0.016	0.016	0.016	0.016	0.016
*P. gingivalis* BeTR415	0.063	0.063	0.125	0.063	0.063	0.125
*F. nucleatum* BeTa9-1	0.016	0.016	0.016	0.016	0.016	0.016
*F. nucleatum* BeOR1	0.016	Missing	0.250	0.250	0.250	0.250
*F. nucleatum* BeW10	0.063	0.063	Missing	Missing	Missing	Missing
*F. nucleatum* BeFF78	0.063	Missing	0.063	0.063	Missing	Missing

**Table 2:** Used MRM transitions for the LS-determination of minocycline and its internal standard in mouse and rat serum.

Compound	*m*/*z* [M + H]^+^		R_t_ (min)
	Precursor Ion	Product Ion	
Minocycline	458.1	441.3 ^1^	2.12
Minocycline	458.1	352.1 ^2^	
Minocycline-D_7_	465.2	448.3 ^1^	1.99
Minocycline-D_7_	465.2	358.0 ^2^	

^1^ Quantifier, ^2^ Qualifier.

## Data Availability

The raw data supporting the conclusions of this article will be made available by the authors on request.

## Supplementary Materials

The following supporting information can be downloaded at: https://www.mdpi.com/article/10.3390/antibiotics13111012/s1, Figure S1: Representative HPLC-chromatogram after sample preparation for the determination of minocycline in the chamber fluid.; Figure S2: Representative LC-MRM chromatograms of the determination for minocycline in rat serum after the local application of MIN-T.; Figure S3: Visualization of the experimental plan for the experiment using the mouse chamber model; Figure S4: Visualization of the experimental plan for the experiment using the rat periodontitis model.; Table S1: Measured serum concentration of minocycline after the treatment of different dosages of the new formulation into the mouse chamber.; Table S2: Mean values and standard deviations of measurements of the distances and volumes in the trabecular part of the maxilla of rats; Table S3: Mean values and standard deviation of collagen abundance in different parts of the maxilla (trabecular and compact parts) of rats.
