# Supplementary material for: Preclinical Validation of MIN-T: A Novel Controlled-Released Formulation for the Adjunctive Local Application of Minocycline in Periodontitis
Source: Antibiotics (Basel). 2024 Oct 28;13(11):1012. doi: 10.3390/antibiotics13111012 (PMC11591261; doi:10.3390/antibiotics13111012)
Supplement: Supplementary file 1 [file antibiotics-13-01012-s001.zip › antibiotics-3209715-supplementary.pdf]

Supplement

Preclinical Validation of MIN-T: a Novel Controlled-Released Formulation for the Adjunctive Local Application of Minocycline in Periodontitis

Małgorzata Benedyk-Machaczka[1,2], Piotr Mydel[1,2], Karsten Mäder[3], Marta Kaminska[2], Nadine Taudte[4], Marcel Naumann[5], Martin Kleinschmidt[5], Sandra Sarembe[6], Andreas Kiesow[6], Sigrun Eick[7], Mirko Buchholz[4*]

Supporting material:

Figures:

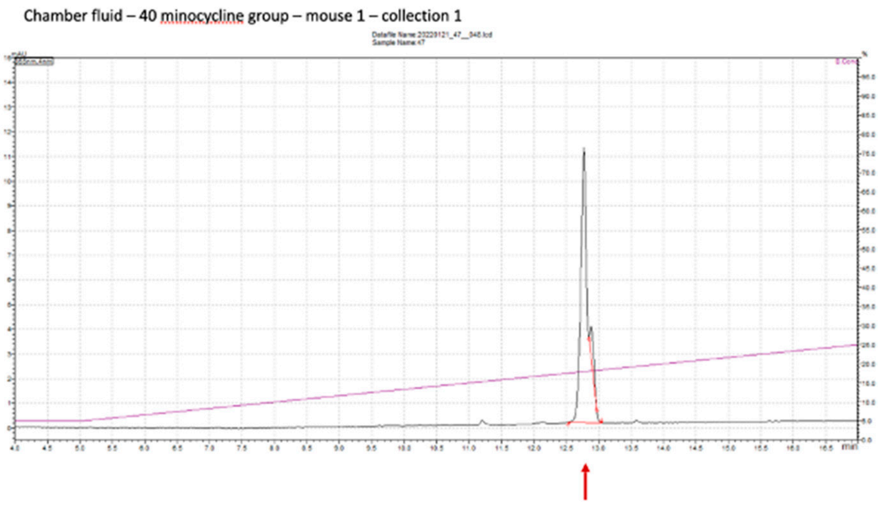

Figure S1: Representative HPLC-chromatogram after sample preparation for the determination of minocycline in the chamber fluid. Here the dosage was 40 mg/kg bodyweight. The calculation of concentration was based on a calibration curve using the AUC.

Supplement

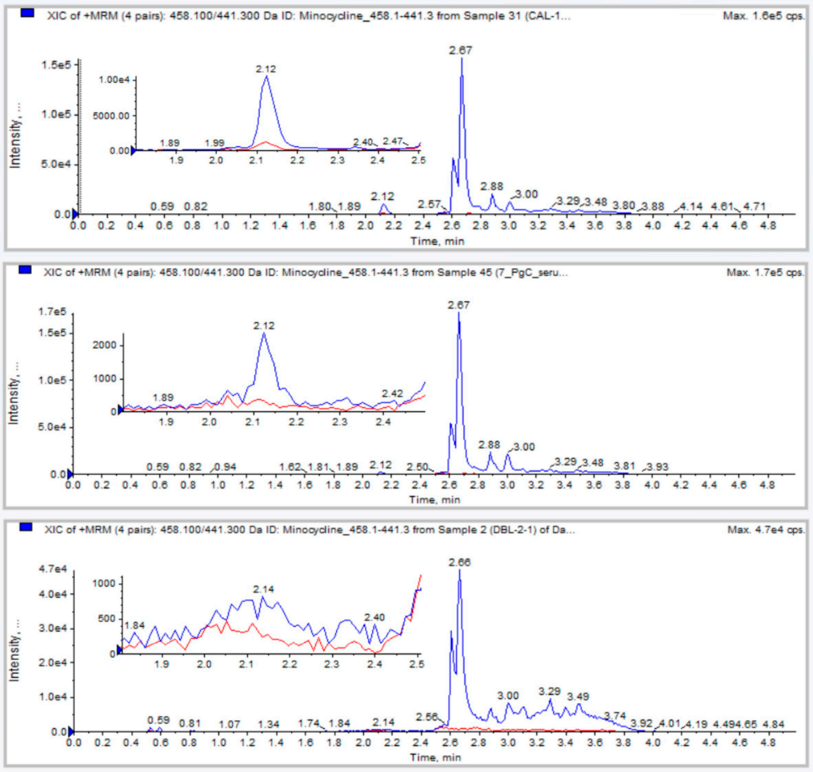

1

Figure S2: Representative LC-MRM chromatograms of the determination for minocycline in rat serum after the local application of MIN-T with a final concentration for minocycline of 20 mg/kg bodyweight, above: calibration standard with 10 ng/ml, central: serum sample from animal 7, below: double blank, retention times: minocycline = 2.12 min, 4-epiminocycline = 2.27 min, minocycline-D7 = 1,99 min (internal standard). As described in the text, the level of systemic minocycline is clear far below the lower limit of quantification (compare upper and middle image)

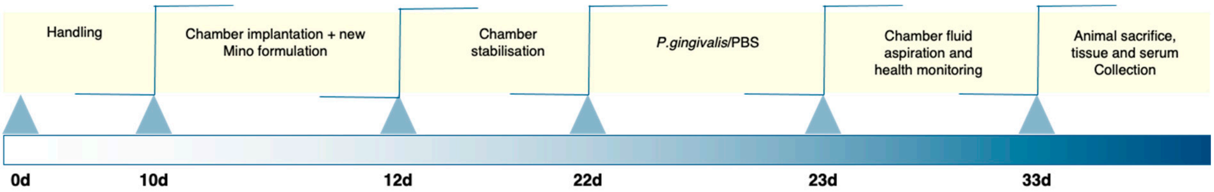

Figure S3: Visualization of the experimental plan for the experiment using the mouse chamber model

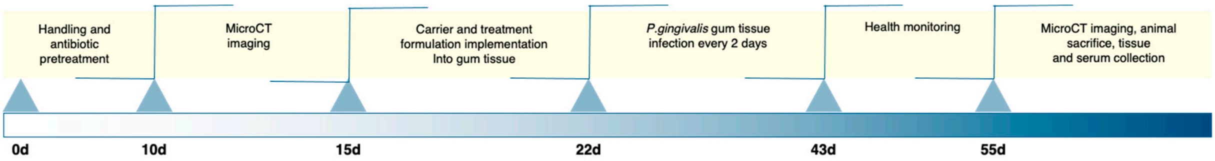

Figure S4: Visualization of the experimental plan for the experiment using the rat periodontitis model

Supplement

Tables:

Table S1: Measured serum concentration of minocycline after the treatment of different dosages of the new formulation into the mouse chamber. Data are collected after the animals were sacrificed. All data shown not statistically relevant, because they are below the original reached LLOQ of 20 ng/ml. They were included to show, that some amount of the compound reaches the systemic circulation after local application at least in the higher dosages of 80 and 40 mg/kg bodyweight.

Dosage	Sample	[c] Minocycline (ng/ml)
80 mg/kg	1	6.7
	2	8.4
	3	7.4
	4	7.1
	5	10.8
	6	9.9
	7	5.0
40 mg/kg	1	7.0
	2	9.7
	3	6.2
	4	8.1
	5	4.2
	6	7.5
	7	5.8
25 mg/kg	1	0.5
	3	1.9
	4	1.7
	5	4.0
	6	4.6
10 mg/kg	1	< 0
	2	1.0
	3	< 0
	4	< 0
	5	< 0
	6	< 0
	7	< 0
1 mg/kg	1	< 0
	2	< 0
	3	< 0
	4	0.5
	5	1.0
	6	1.1
	7	< 0
	8	< 0

Table S2: Mean values and standard deviations of measurements of the distances and volumes in the trabecular part of the maxilla of rats. Values were measured after staining of the tissues. Measurements were done using toluidine blue stained slices of the 1st, 2nd and 3rd molar regions. **BV/TV (%)** trabecular bone volume of the maxilla, **Tb.Th** trabecular thickness, **Tb.N** trabecular number, **Tb.Sp** trabecular space, Groups: **CG** control group, **PG** periodontitis group, **PG+MIN-T** periodontitis+MIN-T treated group,

Parameter	CG	PG	PG+MIN-T
BV/TV (%)	78.19 ± 1.60(11.74)[a]	75.47 ± 1.85(13.23)[ab]	72.16 ± 1.40(10.26)[b]
Tb.N (mm^{-1})	10.73 ± 0.45(3.34)[a]	10.17 ± 0.52(3.71)[a]	11.14 ± 0.42(3.10)[a]
Tb.Th *mean* (μm)	85.48 ± 6.40(47.00)[a]	93.17 ± 8.94(63.85)[a]	72.00 ± 4.16(30.55)[a]
Tb.Th *max* (μm)	130.65 ± 6.93(50.95)[a]	135.81 ± 8.79(62.77)[a]	121.42 ± 7.34(53.95)[a]
Tb.Sp *mean* (μm)	29.12 ± 3.27(24.01)[a]	32.11 ± 2.49(17.81)[ab]	37.91 ± 2.73(20.03)[b]
Tb.Sp *max* (μm)	66.44 ± 6.32(46.42)[a]	69.72 ± 5.41(38.60)[a]	90.40 ± 6.13(45.02)[b]

numbers represent the mean values ± standard error (standard deviation), a, b - mean values between groups labeled with different letters differ significantly ($p \leq 0.05$)

Table S3: Mean values and standard deviation of collagen abundance in different parts of the maxilla (trabecular and compact parts) of rats. Measurements were done using picrosirios red stained slices of the 1st, 2nd and 3rd molar regions. **%Y** percentage of young, poorly-mineralized fine collagen fibrous, **%M** percentage of maturated, mineralized fine collagen fibrous, **(Y/M)** ratio of young (%Y) to maturated (%M) fine collagen fibrous, Groups: **CG** control group, **PG** periodontitis group, **PG+MIN-T** periodontitis+MIN-T treated group.

Parameter	CG	PG	PG+MIN-T
$\%Y_{trabecular}$	0.14 ± 0.06(0.44)[a]	0.28 ± 0.07(0.48)[b]	0.06 ± 0.02(0.18)[a]
$\%M_{trabecular}$	73.19 ± 1.58(11.58)[a]	64.21 ±1.42(10.16)[b]	70.49 ± 1.66(12.21)[a]
$(Y/M)_{trabecular}$	0.002 ± 0.001(0.007)[a]	0.005 ± 0.001(0.010)[b]	0.001 ± 0.0003(0.002)[a]
$\%Y_{compact}$	1.78 ± 0.28(2.37)[ab]	2.53 ± 0.49(4.18)[a]	1.05 ± 0.27(2.27)[b]
$\%M_{compact}$	62.72 ± 1.82 (15.43)[a]	69.98 ± 1.74(14.79)[b]	69.67 ± 1.81(15.39)[b]
$(Y/M)_{compact}$	0.041 ± 0.009 (0.077)[ab]	0.044 ± 0.010(0.083)[a]	0.028 ± 0.010(0.085)[b]

numbers represent the mean values ± standard error (standard deviation), a, b - mean values between groups labeled with different letters differ significantly ($p \leq 0.05$)
